# Development and evaluation of an interoperable natural language processing system for identifying pneumonia across clinical settings of care and institutions

**DOI:** 10.1093/jamiaopen/ooac114

**Published:** 2022-12-30

**Authors:** Alec B Chapman, Kelly S Peterson, Elizabeth Rutter, Mckenna Nevers, Mingyuan Zhang, Jian Ying, Makoto Jones, David Classen, Barbara Jones

**Affiliations:** Informatics, Decision-Enhancement and Analytic Sciences (IDEAS) Center, Veterans Affairs (VA) Salt Lake City Health Care System, Salt Lake City, Utah, USA; Division of Epidemiology, University of Utah School of Medicine, Salt Lake City, Utah, USA; Department of Population Health Sciences, University of Utah School of Medicine, Salt Lake City, Utah, USA; Informatics, Decision-Enhancement and Analytic Sciences (IDEAS) Center, Veterans Affairs (VA) Salt Lake City Health Care System, Salt Lake City, Utah, USA; Division of Epidemiology, University of Utah School of Medicine, Salt Lake City, Utah, USA; Veterans Health Administration Office of Analytics and Performance Integration, Washington, District of Columbia, USA; George E. Wahlen Veterans Affairs (VA) Medical Center, Salt Lake City, Utah, USA; Emergency Physicians Integrated Care (EPIC, LLC), Salt Lake City, Utah, USA; Division of Epidemiology, University of Utah School of Medicine, Salt Lake City, Utah, USA; Department of Population Health Sciences, University of Utah School of Medicine, Salt Lake City, Utah, USA; Data Science Service, University of Utah, Salt Lake City, Utah, USA; Department of Internal Medicine, University of Utah School of Medicine, Salt Lake City, Utah, USA; Informatics, Decision-Enhancement and Analytic Sciences (IDEAS) Center, Veterans Affairs (VA) Salt Lake City Health Care System, Salt Lake City, Utah, USA; Division of Epidemiology, University of Utah School of Medicine, Salt Lake City, Utah, USA; Division of Epidemiology, University of Utah School of Medicine, Salt Lake City, Utah, USA; Informatics, Decision-Enhancement and Analytic Sciences (IDEAS) Center, Veterans Affairs (VA) Salt Lake City Health Care System, Salt Lake City, Utah, USA; Division of Pulmonary & Critical Care Medicine, University of Utah School of Medicine, Salt Lake City, Utah, USA

**Keywords:** natural language processing, pneumonia, diagnostic errors, quality indicators, health care, electronic health records

## Abstract

**Objective:**

To evaluate the feasibility, accuracy, and interoperability of a natural language processing (NLP) system that extracts diagnostic assertions of pneumonia in different clinical notes and institutions.

**Materials and Methods:**

A rule-based NLP system was designed to identify assertions of pneumonia in 3 types of clinical notes from electronic health records (EHRs): emergency department notes, radiology reports, and discharge summaries. The lexicon and classification logic were tailored for each note type. The system was first developed and evaluated using annotated notes from the Department of Veterans Affairs (VA). Interoperability was assessed using data from the University of Utah (UU).

**Results:**

The NLP system was comprised of 782 rules and achieved moderate-to-high performance in all 3 note types in VA (precision/recall/f1: emergency = 88.1/86.0/87.1; radiology = 71.4/96.2/82.0; discharge = 88.3/93.0/90.1). When applied to UU data, performance was maintained in emergency and radiology but decreased in discharge summaries (emergency = 84.7/94.3/89.3; radiology = 79.7/100.0/87.9; discharge = 65.5/92.7/76.8). Customization with 34 additional rules increased performance for all note types (emergency = 89.3/94.3/91.7; radiology = 87.0/100.0/93.1; discharge = 75.0/95.1/83.4).

**Conclusion:**

NLP can be used to accurately identify the diagnosis of pneumonia across different clinical settings and institutions. A limited amount of customization to account for differences in lexicon, clinical definition of pneumonia, and EHR structure can achieve high accuracy without substantial modification.

## INTRODUCTION

Electronic health records (EHRs) contain detailed information that can be leveraged to improve patient care and research.^1^ However, because its primary purpose is to support clinical work, using the EHR for system-level measurement is challenged by missing data, inaccurate documentation, and lack of standardized structured formats.^2–5^

Natural language processing (NLP) can extract relevant data from clinical text to enhance system-level measurement. Examples include adverse events,^6–12^ social determinants of health,^13–17^ and infectious disease surveillance.^18–24^ However, a major barrier to using NLP for research is interoperability. Few NLP systems are designed to generalize beyond a single institution to different EHRs or documentation practices.^25^^,^^26^ Additionally, even within institutions, the vocabulary and structure of a clinical note vary across clinical settings with different types of notes.^27^ Thus, many tools decrease in performance when transported to a new setting, preventing continuous use and refinement of previous work.

The problem of NLP interoperability is well illustrated in the domain of pneumonia. As the leading infectious cause of death in the United States,^28–30^ pneumonia is the target of multiple quality improvement and research efforts. Inappropriate diagnosis can lead to overuse of antibiotics,^31^ or delayed treatment.^32^ Most system-level measures of pneumonia rely on diagnosis codes, which are inaccurate and variable.^33–37^ To fill this gap, multiple NLP tools have been developed to extract pneumonia from clinical notes.^38–45^ However, previous studies have all focused on a single clinical setting or note type and EHR system and are either proprietary or lack the information necessary to be implemented, tested, or refined within different systems.

In this work, we aimed to develop an open-source, usable, flexible, and generalizable NLP approach for extracting assertions of pneumonia across different clinical settings and institutions to improve the measurement of pneumonia. To create a robust system, we used a diverse sample of annotated notes from the Department of Veterans Affairs (VA) EHR and designed our system to extract assertions of pneumonia from 3 types of clinical notes: emergency department notes (ED), chest imaging radiology reports (RAD), and discharge summaries (DC). To evaluate the interoperability of this method, we then evaluated and refined our system with EHR data from the University of Utah (UU).

## MATERIALS AND METHODS

### Dataset and participants

We identified patients ≥18 years who were hospitalized from the emergency department from January 1, 2015 to April 30, 2021 in VA or UU. The VA healthcare system is the largest integrated healthcare system in the United States, with 161 emergency departments, a shared EHR (VistA), and a clinical data warehouse that contains granular data at the national level. The UU consists of a single academic hospital-affiliated emergency department and one additional emergency department.^46^ We retrieved data from the UU Enterprise Data Warehouse (EDW), which is collected from the UU EHR (Epic).

Inclusion criteria and sampling procedure were similar in both systems (Supplementary Materials) and included keyword searches, document titles, and signing provider specialties. Documents were selected using simple random sampling over all eligible notes in each institution and were randomly assigned to a development set and testing set.

### Annotation

Two annotators (an emergency physician [ER] and a pulmonary and critical care physician [BJ]) annotated notes and developed consensus guidelines (Supplementary Materials). For each note type, annotators iteratively reviewed identical batches of 10–20 notes, measured inter-annotator agreement (IAA), and resolved disagreements. Once an IAA of Cohen’s Kappa ≥ 0.8 was reached,^47^ the annotators single-annotated sampled notes from each setting for the development sets and double-annotated additional documents for the testing set. Final IAA was measured on each testing set.

Annotators classified each document as either “Positive,” which included both definite and possible diagnoses of pneumonia, or “Negative.” Definitions of a pneumonia diagnosis varied by setting according to the different document structures and lexicon. Complete setting-specific classification schemas are described in the Supplementary Materials.

### NLP system

#### High-level design

We designed our NLP system with the intent of supporting multiple use cases. One was to enable detection of pneumonia in any of the 3 individual note types for cohort creation or disease surveillance. A second was to enable population-level studies that examine pneumonia diagnostic discordance, defined as a difference between the initial diagnosis in the ED and later diagnoses from the chest imaging report or discharge summary. A third use case was to enable provider-specific feedback for discordant cases.

**Figure 1.**
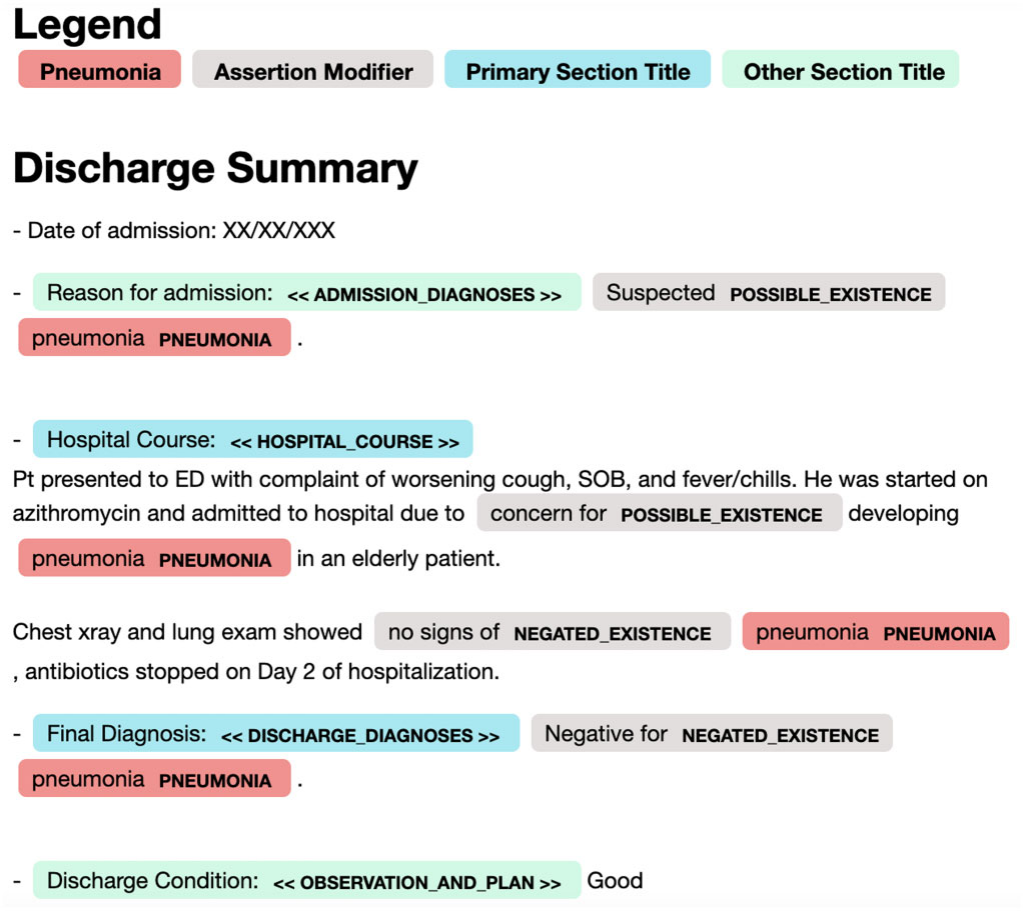
Visualization of a discharge summary documenting a case of misdiagnosed pneumonia. Pneumonia is first documented in the “Admission Diagnosis” section, indicating this was the diagnosis from the emergency physician, whereas the “Discharge Diagnosis” section lists CHF as the final diagnosis. This is explained further in the “Hospital Course,” where the author explains that pneumonia was ruled out after the patient was admitted. Mentions of pneumonia extracted by the NLP and associated linguistic modifiers are labeled accordingly and highlighted in red. Sections are labeled using normalized section titles, and sections considered relevant for document classification are highlighted in blue, while other sections are in green.

**Figure 2.**
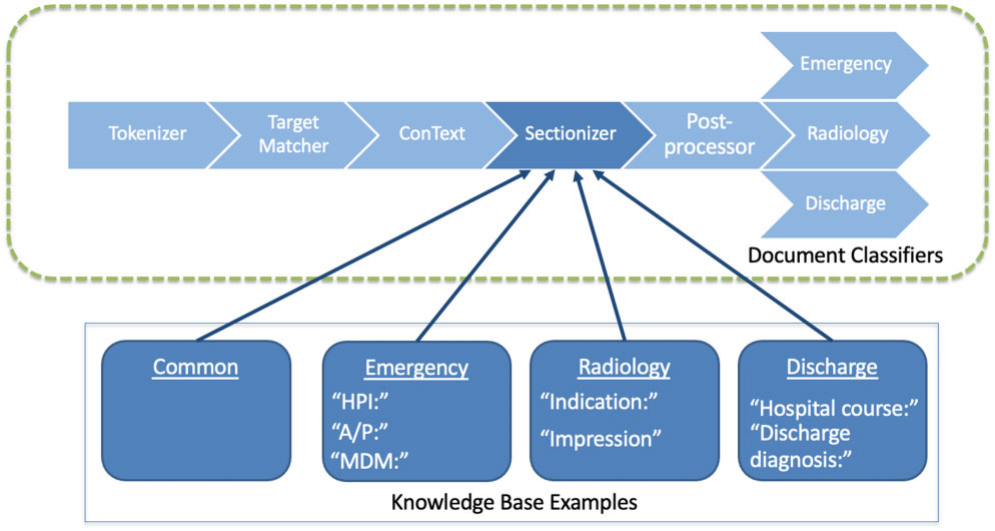
Conceptual representation of the modular NLP system design.

These multiple use cases necessitated a flexible, customizable architecture. Accordingly, we designed the system as a modular rule-based pipeline, allowing us to maintain a shared ontology and primary logic consistent across all settings while accounting for the variation in lexical content and clinical definitions across settings, use cases, and institutions. We implemented the system using the Python package medspaCy.^48^ An example of a discharge summary processed by our system is shown in Figure 1. The overall design of our pipeline is shown in Figure 2. All code necessary for implementation, along with examples of rules and detailed explanations of each component, is publicly available on GitHub (https://github.com/abchapman93/medspacy_pneumonia).

Customization also allowed prioritization of different performance characteristics for each clinical setting to match the intended purpose for each note type. For example, since the standard of care for pneumonia is to confirm a clinical diagnosis by chest imaging, the primary purpose of a radiology report is to confirm positive clinical diagnoses of pneumonia.^49^ As such, we tailored the RAD system to prioritize recall over precision.

#### Entity extraction

Following initial standard processing steps such as tokenization, part-of-speech tagging, and dependency parsing, the first step in our system was entity extraction using semantic and syntactic patterns within medspaCy’s *TargetMatcher* component. Examples of concept labels included “pneumonia,” “opacification,” and “anatomy.” A complete list of terms is provided in the Supplementary Materials and on GitHub.

Some examples of setting-specific entity rules were “airspace disease” and “infectious process.” These phrases were included in the RAD pipeline because they have specific meaning in this setting but were excluded from the ED and DC pipelines as they were ambiguous in clinical notes. Additionally, the RAD pipeline extracted concepts pertaining to alternate diagnoses such as atelectasis and pulmonary edema.

#### Attribute detection

**Figure 3.**
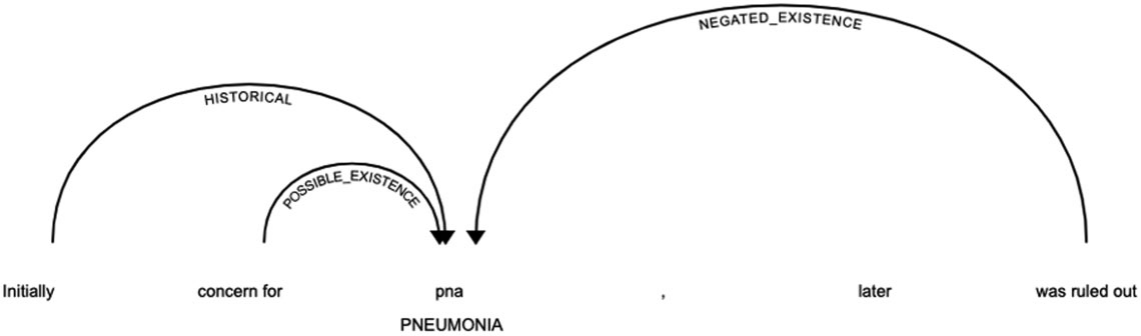
The ConText algorithm detecting linguistic attributes for a mention of pneumonia. Arrows directed from modifiers to entities indicate that the modifiers are linked to the entity. Here, a mention of pneumonia is linked to the historical modifier “initially” (referring to an earlier part of the hospitalization), the uncertainty modifier “concern for,” and the negation modifier “was ruled out.” This mention of pneumonia would thus be considered “historical,” “uncertain,” and “negated.”

The *ConText* algorithm identified relevant attributes including negation, uncertainty, temporality, and hypothetical assertion (ie, not directly related to the patient’s current diagnosis; for example, “return to the ED **if** you experience symptoms concerning for pneumonia”) from neighboring text.^50^ An example of *ConText* is shown in Figure 3.

Some phrases which may be used to assert a possible diagnosis in the clinical pipelines needed to be handled differently in the RAD pipeline. For example, in an ED note, the phrase “rule out pneumonia” in the Assessment/Plan indicates that the author believes a diagnosis of pneumonia is likely enough to merit evaluation. In this case, we considered this to be a possible diagnosis. However, in a chest imaging report, the phrase “rule out” pneumonia is used as an indication for the procedure rather than the radiologist’s diagnosis. In this context, “rule out” was treated as hypothetical and would not merit a positive classification.

#### Section detection

We used medspaCy’s *Sectionizer* component to identify sections in the texts. This component identified headers in the text which denote the beginning of a new section (eg, “Past Medical History,” “Medical Decision Making,” and “Assessment/Plan”) and then assigned a normalized section category to each entity in that section. Since the annotation guidelines explicitly refer to different sections of a note for each domain, identifying which section of a note contained a mention of pneumonia was crucial for correctly classifying each note. Because the VA EHR does not enforce standardized note templates, there were many different variations of section titles that needed to be captured in the pipeline. For example, “Active Problem List:,” “Secondary Diagnoses and Co-morbidities,” and “Computerized Problem List is the source for the following:” all needed to be mapped to the category of “Problem List.” In some contexts, section titles could be ambiguous, such as “Impression:” in an emergency note, which could refer either to the Impression section of an imaging report pasted into the note or to the Assessment/Plan.

#### Postprocessing

The medspaCy *Postprocessor* component executes custom business logic on the entities in a document. These rules dealt with ambiguous terms or updating attributes based on other findings in the note. One important postprocessing rule disambiguated “CAP” in clinical notes as meaning either “community-acquired pneumonia” or referring to a form of medication. Another postprocessing rule ignored irrelevant mentions of opacification in radiology reports by linking them to an anatomical site (eg, “suboptimal ***opacification*** of the pulmonary artery”). Another rule differentiated between “Impression” sections from embedded radiology reports and Assessment/Plan sections in emergency notes.

#### Document classification logic

A custom document classifier component was implemented separately for each domain. Each document classifier attempted to replicate the logic specified in the annotation guidelines based on the entities and corresponding attributes in the document. The primary difference between classification logic for ED and DC notes was which sections were considered for a “Positive” classification. The radiology system included radiographic concepts in classification logic and attempted to exclude findings supporting a diagnosis other than pneumonia.

##### Customization for generalizability

VA and UU use different EHRs (VistA and Epic, respectively) and have different note structures. It was therefore necessary to modify the NLP system to match the UU note structure. However, we hypothesized that the NLP could be adapted to the new institution with only a small amount of customization in the NLP design. To implement these customizations, we created a separate set of knowledge base files for UU data. Most additional resources were additional section headers which matched the structure of a small sample of UU development notes, as well as some rules to match entity terms or modifiers which were not seen in VA notes. We did not modify the document classification logic.

### Machine learning comparison

To explore the feasibility and performance of machine learning approaches, we fine-tuned and evaluated a pre-trained BERT Transformer model.^51^ Annotated documents from the 3 VA development sets were each split into separate training and validation sets. The BERT model was fine-tuned on the training set, and the validation set was used to choose values of hyperparameters including number of epochs, learning rate, and weight decay were evaluated using a random walk on the validation dataset. The Transformer model enforced a constraint that documents had a maximum length of 512 subword tokens. For documents exceeding this limit, we removed text following the last keyword related to pneumonia. No additional preprocessing (eg, section splitting) was performed for the Transformer model.

### Evaluation

We next evaluated the NLP’s performance at identifying pneumonia in each note type using the testing sets from each institution. Using human annotation as the gold standard, we measured binary classification performance of using precision, recall, and F1 (Precision = (# TP)/(# TP + # FP); Recall = (# TP)/(# TP + # FN); F1 = 2*Precision*Recall/(Precision + Recall); TP = true positives; FP = false positives; FN = false negatives) of (1) the rule-based system in VA data; (2) the Transformer model in VA data; (3) the “out-of-box” rule-based system in UU data with no modifications (to test how well our system could generalize to a new system without additional development effort); and (4) the customized rule-based system in UU data.

### Error analysis

We conducted error analysis among all testing documents with an error in any of evaluations (1), (3), and (4). Each case was reviewed by an NLP developer (ABC) to identify sources of NLP error, and cases with ambiguity were additionally reviewed by a clinician (BJ). We identified general categories of errors and tabulated the total number of errors by category in each evaluation.

## RESULTS

### Dataset and patient cohort

Summary statistics for counts of patients in both cohorts and relevant notes are displayed in Table 1.

**Table 1.** Summary statistics for our dataset and cohort in both institutions

	VA	UU
**Entire cohort**		
Date range of hospitalizations	January 1, 2015–April 30, 2021	January 1, 2015–April 30, 2021
Count of hospitalizations in cohort	2 203 165	74 601
Gender		
Male	2 082 995 (94.6%)	39 699 (53.2%)
Female	120 153 (5.4%)	34 898 (46.8%)
Missing	17 (<0.1%)	4 (<0.1%)
Race		
White	1 554 480 (70.56%)	58 931 (79.0%)
Black or African American	511 124 (23.20%)	1953 (0.03%)
American Indian or Alaska Native	16 381 (0.74%)	1445 (2.62%)
Native Hawaiian or other Pacific Islander	14 940 (0.68%)	1347 (1.8%)
Asian	10 036 (0.46%)	1506 (2.02%)
Missing/other	96 204 (4.37%)	9419 (12.6%)
Age		
18–29	19 949 (0.91%)	8729 (11.7%)
30–39	71 614 (3.25%)	9856 (13.2%)
40–49	102 738 (4.66%)	10 207 (13.7%)
50–59	288 803 (13.11%)	13 190 (17.7%)
60–69	675 666 (30.67%)	14 309 (19.2%)
70–79	628 205 (28.51%)	10 364 (13.9%)
80–89	305 321 (13.86%)	6315 (8.5%)
Over 90	110 852 (5.03%)	1631 (2.2%)
Missing/other	17 (<0.1%)	0 (0%)
Total count of notes with keywords		
Emergency	1 219 868	18 931
Radiology	1 846 246	82 051
Discharge	2 130 274	10 506
**Annotated corpus**		
Counts of notes^a^ (% positive)		
Emergency	300 (38.0%)	195 (49.7%)
Radiology	500 (28.9%)	187 (44.4%)
Discharge	300 (48.0%)	185 (45.4%)
Mean document character lengths (SD)		
Emergency	8136 (5168)	17 042 (6731)
Radiology	861 (751)	969 (586)
Discharge	8370 (4809)	12 473 (5954)

a 100 notes of each type in each institution were belonged to the testing set.

### Annotation

For the VA development set, clinicians annotated a total of 800 documents (200 ED notes, 400 RAD reports, and 200 DC summaries). For the VA testing set, 300 notes were double-annotated with disagreements reviewed and resolved by consensus. The final IAA on the testing set was Cohen’s Kappa = 89.9 for ED, 94.9 for RAD, and 83.9 for DC.

For the UU dataset, clinicians annotated a total of 267 documents for development, with 91 double-annotated to measure agreement (Kappa = 0.89). One clinician (BJ) then annotated 100 documents from each setting for testing. UU testing notes were not double-annotated, as the high IAA established confidence in annotator reliability using the UU dataset.

### NLP evaluation

A total of 782 rules were included in our VA pipeline (307 target concept rules, 288 section rules, 178 context rules, and 9 postprocessing rules). Within the VA testing data, the rule-based system achieved high precision in ED and DC, moderate precision in RAD, and high recall in all 3 settings (Table 2). Precision and recall were fairly balanced in both ED and DC while recall was higher for RAD, matching our objectives for prioritizing precision or recall in each setting. The Transformer model demonstrated lower precision for ED, lower recall for RAD and DC, slightly higher recall for ED, and higher precision for DC.

**Table 2.** NLP performance in the VA testing set (*n* = 100 documents per note type) for rule-based and the Transformer model

	Rule-based system			Transformer model		
VA	Precision	Recall	F1	Precision	Recall	F1
Emergency	88.1^a^	86.0	87.1^a^	45.8	88.4^b^	60.3
Radiology	71.4^b^	**96.2** ^a^	82.0^a^	66.7	69.2	67.9
Discharge	**88.3**	93.0^a^	**90.1** ^b^	**93.2** ^b^	71.9	**81.2 **
Macro Avg	83.9	91.2	87.4	68.6	76.5	69.8

*Note*: For each model, the highest value of each metric is shown in bold.

a The model outperformed the comparison by at least 10 percentage points.

b The model outperformed the comparison in that particular metric for that note type.

Within the UU testing data, the overall performance of the VA-developed NLP “out-of-box” yielded quite similar performance to VA, with the exception of discharge summaries (Table 3). Recall was high in all domains, increasing for both ED and RAD and dropping only slightly for DC. Precision increased in RAD and saw a small decrease for ED. The largest drop in performance was precision for DC. Customizing the NLP for UU data with 34 additional rules improved performance for all note types and metrics and achieved generally higher performance than in VA, with the exception of precision and F1 for DC.

**Table 3.** NLP performance using UU data (*n* = 100 documents per note type). The highest performance in each column is shown in bold

	UU out-of-box			UU customized		
	Precision	Recall	F1	Precision	Recall	F1
Emergency	**84.7**	94.3	**89.3**	**89.3** ^a^	94.3	91.7^a^
Radiology	79.7	**100.0 **	87.9	87.0^a^	**100.0**	**93.1** ^a^
Discharge	65.5	92.7	76.8	75.0^a^	95.1^a^	83.4 ^a^
Macro Avg	76.7	95.7	85.2	84.0	96.5	89.8

*Note*: “UU Out-of-Box” performance using the exact system developed in VA, while “UU Customized” was customized for UU data.

a The customized system outperformed the out-of-box system in this metric.

### Error analysis

**Figure 4.**
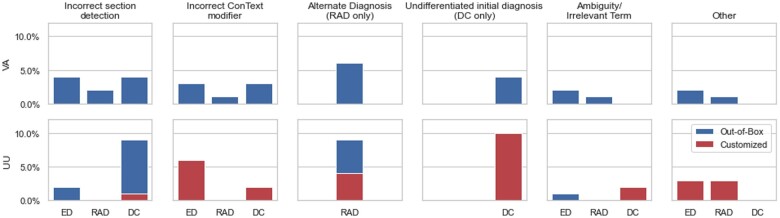
A plot showing the counts of NLP errors by category in both the VA and UU. The categories correspond to those defined in Table 4. The counts of errors in emergency, radiology, and discharge notes are displayed in clusters within each error category. The top plot shows counts in VA, while the bottom plot displays counts in the UU out-of-box evaluation in blue and the remaining counts of errors after customization in red. Completely red bars indicate that the errors were not reduced after customization.

Within the 600 testing documents, we identified a total of 80 NLP errors, consisting of 33 in VA and 47 in out-of-box UU; 31 of the original UU errors remained after customization, and no new errors were introduced by customization. Descriptions and examples of each error type are shown in Table 4. Figure 4 displays the counts of errors by categories category within each domain and institution.

**Table 4.** Descriptions and examples of errors found during error review

Error category	Description and examples
Incorrect section detection	A section header was not recognized by the Sectionizer component, a section header was misclassified as denoting a different section category, or there was no section header in the note. “**DISCHARGE DIAGNOSIS AND BRIEF EXPLANATION:**”—Not recognized as *Final Diagnosis* in Discharge Summary “**Clinical statement: Pneumonia**”—Not recognized as *Reason for Exam* in radiology report “**A- Pneumonia**”—Not recognized as *Assessment/Plan* “**Patient Active Problem List Diagnosis**”—Section title in UU not included in out-of-box implementation
Incorrect ConText modifier	A linguistic modifier was either incorrectly identified, incorrectly linked to an entity, or failed to be linked to an entity. “**recent** Covid infection, super-imposed **pneumonia**?”—“recent” distributed to “pneumonia” and marked as *historical* “Had **pna in early Feb”**—not recognized as *historical* “Chest x-ray **was negative pna**”—“was negative” linked to entities earlier in the sentence but not after
Ambiguity/ irrelevant term	A phrase was erroneously identified as referring to a diagnosis of pneumonia. “**infectious process** (osteomyelitis)”—infectious process incorrectly identified as *Pneumonia* in a radiology report “Is given strict **pneumonia injections**”—not recognized as referring to a pneumonia vaccine
Alternate diagnoses (radiology only)	In a radiology report, a finding such as *opacity* or *consolidation* was referring to a different diagnosis such as *atelectasis* but was attributed to pneumonia by the NLP. “Left perihilar **opacity** is again noted. Mild **atelectasis** is present.” “Findings: mild basilar **opacities**… Impression: likely **atelectasis**” “Chronic airspace **opacity**”
Undifferentiated initial diagnosis (discharge only)	In a discharge summary, the clinical narrative discusses the initial diagnosis which is incorrectly classified as the discharge diagnosis by the NLP. “Upon **admission**, there was concern for autoimmune disease and **pneumonia**” “Pt **arrived to ED** with productive cough, was treated with cough suppressant and abx for **pneumonia**”—ED course included treatment for pneumonia but was not ultimately considered a diagnosis following admission.
Other	Miscellaneous typos, templates, and EHR structure issues. “Signs of **pneumonia** on imaging - 0”

Across all note types, the most common error in both institutions was incorrect section detection, where a section title was either missed due to non-standard nomenclature or assigned the wrong classification. This was most common in clinical notes and was especially prevalent when running the “out-of-box” system in UU.

The most common error for radiology reports was failure to link radiographic findings to an alternate diagnosis, leading to false positives.

For DC summaries in UU, a common source of error for both the “out-of-box” and customized systems was confusion between initial and final diagnoses. Most of these cases were false positives extracted from the “Hospital Course” which were documenting earlier diagnostic possibilities but did not reflect the final diagnosis. As shown in Table 1, notes in UU tended to be longer than in VA, and we found that the “Hospital Course” section in UU typically contained much more background information about a patient’s condition, making it difficult for the NLP to differentiate between the author’s final diagnostic assertion and intermediate diagnoses during the hospitalization.

Other issues seen in each of the notes included incorrect modifier linking, ambiguous terms, patient instructional text, and some cases of annotation error.

## DISCUSSION

### Key findings

Using clinical texts from 2 healthcare and EHR systems, we developed and evaluated a flexible, generalizable NLP system that can accurately identify diagnostic assertions of pneumonia across different clinical settings and institutions. We found that high performance could be achieved in a new institution by customizing the system using a small number of addition rules to account for EHR structural differences found in a small set of annotated documents. Performance was particularly high for chest imaging and emergency department notes, even without modification, suggesting that interoperable NLP tools for identifying pneumonia within multiple institutions are feasible.

We primarily used rule-based rather than machine learning methods. Surveys comparing rule-based to machine learning approaches have shown several practical advantages to rule-based NLP, including interpretability, the ability to incorporate domain knowledge, the relatively simple process of modifying rules for new use cases and datasets, and the smaller amount of labeled data required for training.^52^ Due to the manual effort needed to annotate data available, as well as limited computational resources, rules allowed us to achieve high performance by leveraging human expertise. The comparison with a pretrained Transformer model demonstrated that our system achieved higher performance in most metrics than a machine learning model trained on the same dataset.

A rule-based design also supported our goal of building a system that was usable across different settings and institutions. The modifiable nature of rules allowed us to easily update the system to fit new data. Adapting machine learning models to new settings requires retraining or fine-tuning with sufficiently large sets of annotated data. We achieved high performance in UU while annotating only one-third of the number of documents used in VA development, requiring approximately 22 h less clinician time (based on an estimated annotation time of 2–3 min per document). Furthermore, trained machine learning models are not easily transported between institutions due to privacy concerns when they are trained on sensitive medical data.^53^ Rule-based models, on the other hand, do not suffer from this limitation, and can be shared and reused easily and safely.

An additional advantage of using a rule-based system was explainability. One potential use of our system is to provide feedback to clinicians regarding cases in which diagnoses were discordant across a hospitalization. As the logic of rules can be clearly scrutinized by users, this approach helps users to understand the underlying logic of the system,^54^^,^^55^ which is essential for building transparency and trust among clinicians during feedback or decision support.^56^ Some machine learning methods are interpretable, and the field of explainable artificial intelligence has made progress in developing interpretable machine learning tools.^57^ Combining explainable machine learning with rule-based approaches to generate measures that are simultaneously highly accurate, resilient to changes in data, interpretable, and meaningful across multiple institutions is the subject of future work. Additionally, future work will evaluate the feasibility of communicating NLP findings to clinicians for the purpose of performance feedback.

### Related work

Our work builds upon existing studies of NLP in pneumonia but represents several important advances. First, the majority of previous work has focused on extracting radiographic evidence of pneumonia from radiology reports,^38–40^^,^^42–44^ which does not shed much light on clinical diagnoses. Similar to our radiology pipeline, most of these studies achieved moderate precision and higher recall. There are far fewer studies utilizing text from care teams to identify clinical diagnoses. Bejan et al developed a machine learning algorithm to identify pneumonia diagnoses among intensive care unit patients using features extracted from multiple note types and found moderate accuracy at the patient level.^45^ Their study focused on a small sample of patients from a single institution. Jones et al used a combination of structured data and a machine-learning NLP from the VA system to classify initial diagnoses of pneumonia from emergency department notes,^41^ finding that NLP achieved much higher recall than ICD codes and that combining NLP with ICD codes achieved the highest overall score. An important finding of this study was that NLP identified more uncertain cases of pneumonia, which is important when studying the evolution or change in diagnosis for an individual patient across an episode of care. None of the previous studies tested the NLP system outside of the institution in which it was developed or publicly shared the details needed to implement the tool.

### Implications

The findings in this study have several important implications on research and quality improvement within a learning healthcare system. To our knowledge, our study is the first to assess the interoperability of an open-source pneumonia NLP across different clinical settings, institutions, and EHR systems. Interoperability is crucial to development of measures that are used to examine and improve quality. Currently, much of measurement in healthcare relies upon chart review, which is burdensome and variable, leading to a call for electronic clinical quality measures (eCQMs).^58^ In order for eCQMs to be useful, they must reliably represent the same constructs of quality across institutions.

To understand and improve quality of diagnosis, the accuracy and interoperability of the proposed system demonstrate the potential of using NLP to measure the evolution of diagnosis, which can be used for feedback at the both the system and provider level. As the EHR becomes increasingly ubiquitous and powerful in clinical care, we can leverage it to support learning. Since a large amount of EHR data are recorded in clinical text, important elements that are not easily coded, such as diagnosis, can be lost unless we develop automated approaches through NLP. If we are to truly leverage the clinical experience from millions of patient encounters, we must develop NLP methodologies that are simultaneously accurate, interpretable to humans, and designed for meaningful improvement.

### Limitations and future work

We recognize several opportunities to improve our system with further refinements. The existing tool was developed and tested with a limited amount of annotated data from only 2 systems, one of which included a single facility that demonstrated some redundancy in the patient population (10 patients had notes in both the development and testing sets), which raises the possibility of bias in our testing results for the UU. No such overlap occurred in VA.

Because the goal was to identify any diagnosis of pneumonia even if there was uncertainty, we included both definite and possible diagnoses in our definition of “Positive” documents. Differentiating between these 2 types of cases were helpful in defining annotation guidelines, but we ultimately collapsed them into one class for adjudication and NLP classification.

While the performance of the system was generally high, excellent performance across all clinical settings is required for the goal of identifying diagnostic discordance, which involves combining classifications from several steps of document processing, each of which has the opportunity for misclassification. Future work will assess the impact of NLP errors on the measure of diagnostic discordance, as well as the feasibility of additional refinements such as improved section detection or combining NLP with structured data.

The Transformer model was limited, as we were unable to pre-train the BERT model on available VA documents. Such pre-training would likely improve the performance of this comparison, but the environment where the experiment was performed did not include graphics processing units, which would support pre-training and more intensive hyperparameter tuning. Future work could include hybrid approaches using both machine learning models and rules which may yield better performance than reported here, although privacy issues may limit portability.

Future work could extend these methods to additional types of notes across a patient’s care (eg, progress notes, outpatient follow-ups). Similarly, while the scope of this study was limited to the diagnosis of pneumonia, future work could extend this approach to other diseases, particularly those that can mimic pneumonia (eg, heart failure). As our approach is fully public and open source, we hope to engage researchers from other systems in evaluating and refining this system for continuous improvement.

## CONCLUSION

Using clinical text from 2 separate institutions and EHRs, we demonstrated the feasibility, accuracy, and interoperability of a flexible, open source NLP system that extracts assertions of pneumonia diagnoses across different clinical settings, healthcare institutions, and EHR systems. We are currently applying this system to examine the process of pneumonia diagnosis across hospitalizations and to develop measures and tools that support provider and system-level feedback and learning. By unlocking data from clinical text, NLP has enhanced our ability to characterize the process of diagnosis at a large scale. This capability will advance our understanding of the process of diagnosis in healthcare and how to best support diagnostic excellence.

## Supplementary Material

ooac114_Supplementary_Data

## Data Availability

Per IRB requirements, patient-level data from this study cannot be shared. Aggregate-level data may be shared upon request. Code is publicly available on GitHub: https://github.com/abchapman93/medspacy_pneumonia
